# Mr. Ring, in Answer to Dr. Kinglake

**Published:** 1806-08

**Authors:** John Ring


### *To the Editors of the Medical and Physical Journal.*

GENTLEMEN,

IN the last Number of your Journal, Dr. Kinglake expresses his opinion, that the inquirers into the source of the cow-pock, have ascribed this disease to the equine fluid, or the matter of grease, in too unqualified terms; and thinks they are too positive in insisting on this point. While he entertains this opinion, it is no wonder he considers me as peculiarly deserving of his animadversion. He thinks I have been more successful in my endeavours to prove the superiority of vaccine inoculation, than in my endeavours to prove, that vaccine and equine matter are of the same nature, and originate from the same source.

He tells us, that in his view of the possible hazard, of trying what might be the event of inserting the acrid and fetid matter of grease, it would not be warrantable for him to extend the experiment to the human subject. This sentiment is highly honourable to his judgment; particularly as the different experiments which he made on the teats of the heifer seemed to indicate, that there is no very extraordinary danger to be thence apprehended.

He asserts that I have not only adduced some authorities for the practice, which I admit; but that I have also represented it to be "*indisputably safe and efficacious*;" which I cannot admit, till Dr. Kinglake, or some other gentleman, will point out the passage in my publications, in which I have been so unguarded.

Supposing, however, that I maintain both these positions, he asks how it comes to pass, that not a single experiment of this kind has been made in our own country? This question, at the same time that it is a natural one for *Dr. Kinglake* to ask, is a proof how little this point has been the object of his attention; and an additional instance to the many others, in your Journal, and elsewhere, in confirmation of what was long ago remarked by Dr. Jenner, that "it is surprising how many authors write on the subject of vaccination, and how few understand it."

Dr. Kinglake asks how it comes to pass, that not a single experiment of the kind has been made in this country, try, notwithstanding it is the very country, where the idea of this supposed identity of the two fluids originated. Here it is obvious that he is totally ignorant of the great number of experiments of the kind made by Dr. Woodville, Mr. Coleman, Mr. Simmons of Manchester, and others, without success; and by Dr. Loy, Mr. Loy, and Mr. Tanner, with success. Dr. Loy, in particular, succeeded repeatedly; and once in a manner which leaves no room for scepticism itself any longer to find an excuse. He borrowed of a gentleman a clean lancet, and in his presence successfully inoculated one of that gentleman's own cows with virus taken from the heel of one of his own horses. In short, no one who reads Dr. Loy's publication, can harbour the least doubt of the identity of the two fluids, without questioning his veracity also; which has never yet been questioned. The same may be said of Mr. Loy and Mr. Tanner, as well as of the foreign Physicians, Dr. Sacco and Dr. La Font. Dr. Sacco having himself been an opponent of this doctrine, was not likely to have been converted to it by any thing short of ocular demonstration; and his evidence, as well as that of Dr. Loy, is the more satisfactory, because it is the result of experiments made before witnesses.

When Dr. Kinglake opposes the result of his own experiments, which were unsuccessful, to that of the experiments of others which was successful, it is necessary to remind him of the observation of a right reverend prelate, in a case of crim. con.; that positive evidence is much stronger than negative. Four servants having been stationed on a landing place, in order to ascertain whether a gentleman went to a lady's apartments, two of them swore they saw him go into it; and the other two swore they did not see it. There is no difficulty in discovering a probable reason, when we recollect, that the two latter might have been asleep at the time. Besides, we all know, that whether the object of inquiry is gallantry or grease, none are so blind as those who *will not see*.

Dr. Kinglake thinks it probable, that others, as well as himself, may have been deterred from making experiments on the human subject, from an apprehension of the ill consequences that might ensue; but this, as he very justly observes, need not have prevented them from making such experiments on the brute animal. Accordingly we find, by the different instances before-mentioned, and others which might easily be adduced, that it has not prevented them. Dr. Kinglake, however, as if he had never heard or read read one syllable of a subject, which has been so hackneyed for years, and is now become so threadbare, once more betrays his total ignorance of it, by gravely and seriously asking, whether the fact has ever been put to the test of experiment? and with what result?

It would, indeed, in this age of experiment, have been a wonder, if such an important fact had continued, till this time, a subject of conjecture; and not been submitted to the test of examination. To what cause the failure of Dr. Kinglake's experiments is to be ascribed, it is not easy to determine, unless the opinion of some of our countrymen, and of Dr. La Font of Salonica, be well founded, that the heel of the horse is subject to more sorts of grease than one.

In order to strengthen his argument, Dr. Kinglake informs us, that neither the farrier who procured the equine virus for him, nor the carter who assisted him in inserting it, had ever experienced any ill effect from such virus; and that they were therefore inclined to consider it harmless. Such an argument is excusable in the mouth of a farrier, or in that of a carter; but not in that of Dr. Kinglake. Thousands may say the same of the itch, the venereal disease, the small-pox, and the plague. These disorders might therefore be considered as not infectious, did not positive and incontrovertible evidence of the contrary too often occur.

Dr. Kinglake admits, that his experiments on this subject may not have been sufficiently numerous to be decisive; which the observations I have already offered, and others that I shall hereafter offer, prove they are not. He thinks, however, they are sufficient to shake my positive assertion, respecting the identity of the two matters in question; and, indeed, they would shake it, were it, as he considers it to be, gratuitous; and were it not supported by positive evidence, and incontestable facts.

Dr. Kinglake is of opinion, that if my notion of the origin of this disease be well founded, it is injudicious to take matter for inoculating the human subject from the cow, lest its native qualities should be impaired by this transmission; and that we ought to have recourse to the fountain head. But, till he can point out an easier method of discovering this fountain head, or rather fountain heel, than he has hitherto done, he may as well tell the inhabitants of Egypt, that it is injudicious in them to quench their thirst, till they have traced their water to its fountain head, and discovered the source of the Nile.

When Dr. Kinglake can consider my assertion on the subject subject as merely gratuitous, although it was sanctioned by several decisive experiments, noticed by himself, it is no wonder he should consider his own experiments conclusive, although not one of them was, or could be, decisive. A negative is not quite so easily proved.

I agree with him, that the discovery of truth is worthy of the best efforts of philosophy; and that neither prejudice, nor intemperate zeal, should interfere with a dispassionate endeavour, to solve the question at issue. But if Dr. Kinglake is determined to consider the two diseases, excited by vaccine and equine matter as radically distinct, till I have ascertained the contrary by my own experiments, it will probably be long before he becomes a convert. At the same time I beg leave to remark, that I should not altogether despair, were I not convinced of the difficulty of discovering the genuine virus in the *horse*, and that such experiments would interfere too much with other duties which I deem still more important.

I shall here recapitulate some of the various instances, in which the cow-pock, or a disease of the same species, appeared to derive its origin from the horse. Dr. Jenner informs us, in his Inquiry, p. 9, that in the year 1770, John Merret, an under gardener to the Earl of Berkeley, lived as a servant with a farmer in the neighbourhood of Berkeley; and occasionally assisted in milking his master's cows. During this period, some horses belonging to the farm, began to have sore heels, which Merret attended; soon after which, the cow-pox appeared on the cows, and several sores on his hands. This man has frequently been put to the test of variolous inoculation, as well as exposure to the natural infection; but to no purpose. It is therefore concluded, that the disease he had was the cow-pock; or the equine disease of the same nature with the cow-pock.

In p. 22, of the same work, Dr. Jenner relates another case of this kind. In 1782, Simon Nichols, servant of Mr. Bromedge, having been employed in a similar way, the disorder was communicated by him also to his master's cows; and having quitted Mr. Bromedge's service without any sores on him, and gone to another farm, the disorder soon afterwards appeared on his hands; and was there likewise communicated to the cows.

In p. 25, Dr. Jenner relates the case of Thomas Pearce, the son of a smith and farrier, who had a similar disorder, when a lad, from dressing the heels of a horse; and has since proved insusceptible of the infection of the smallsmall-pox, both in inoculation and the natural way. In a note, at the bottom of the next page, he says, " It is a remarkable fact, and well known to many, that we are frequently foiled in our endeavours to communicate the small-pox by inoculation to blacksmiths, who in the country are farriers. They often, as in the above instance, either resist the contagion entirely; or have the disease anomalously. Shall we not be able to account for this now, on a rational principle?"

In p. 26, he relates the case of Mr. James Cole, of Berkeley, who also had this disease from the heels of a horse. When inoculated for the small-pox, a few eruptions appeared on his forehead; but soon disappeared, without maturation.

In p. 27, he relates the case of Mr. Riddiford, of Stone, in the parish of Berkeley, who also had a severe local and constitutional affection from dressing the heels of a horse. In this case, it is true, it may be questioned whether infection was received while the virus in the horse retained its prophylactic power; but either this case, or the former, is sufficient to shew, that equine matter is not inert, as Dr. Kinglake endeavours to prove.

In p. 28, he relates a case of a dairy maid who had the cow-pox; and, in p. 32, informs us, that from this time his researches were interrupted till the spring of the year 1798, when, from the wetness of the early part of the season, many of the horses of the farmers in the neighbourhood were affected with sore heels; in consequence of which the cow-pox broke out in several dairies, which afforded him an opportunity of making further observations on that curious disease. A horse belonging to a person who keeps a dairy, in a neighbouring parish, had sore heels; in consequence of which three men received infection. Two of them, who had the small-pox before, described the constitutional symptoms they felt as similar to those of that disorder; and one of them being employed in milking, the cows also shewed signs of being infected with the genuine cow-pock, in about the space of ten days after he had dressed the heels of the horse.

In p. 33, we are informed, that John Baker was inoculated with matter taken from a pustule on the hand of another of these men, who were thus infected by means of equine matter; and a pustule was thereby excited. This pustule was not exactly similar to those which had been excited by matter from the cow, or the human subject; but it nevertheless furnished another proof that equine quine matter is far from being inert. It is the subject of the second plate contained in Dr. Jenner's Inquiry; which is generally, but erroneously, supposed to represent a case of the cow-pock. Whether it would have afforded a security against the infection of the small-pox, Dr. Jenner was prevented from deciding, the boy having, soon after, felt the effects of a contagious fever in a workhouse.

In p. 44, Dr. Jenner tells us, that he entertains no doubt of the cow-pox originating from the grease; being well convinced, that it never appears among cows, but when they have been milked by some person who has the care of a horse labouring under this disease; unless it has been introduced into the farm, either by a cow, or a servant, already infected.

In page 45, he says, the activity of equine virus is increased by transmission through the cow. This opinion is since proved to be erroneous; but it was the result of such cases as had at that time come under Dr. Jenner's observation. His next remark, however, is consistent with his usual accuracy; for he tells us, the virus in the horse is most active at the commencement of the disease, before it has acquired the consistence of pus; and says, he is not confident whether its infectious quality does not entirely cease, as soon as it is secreted in this form.

He remarks, that it is very easy to procure pus from old sores on the heels of horses; and says, he has *often* inserted such into scratches, made with a lancet, on the nipples of cows, without producing any other effect than simple inflammation. This shews, that the opinion entertained by Dr. Jenner and others, has *not* been entertained without a solitary experiment being made. The experiments made by him, it is true, did not confirm the opinion; but it has been fully confirmed by the experiments of others, since made. He seems, however, to have been perfectly right in his conjecture, that it is the thin fluid, of a darkish colour, oozing from the recent cracks in the heels of a horse, which communicates the disease in question.

In p. 62, he relates a case, which renders it probable, that not only the heels of the horse, but also other parts of the animal, are capable of generating the virus which produces the cow-pox. An extensive inflammation, of the erysipelatous kind, took place, on the upper part of the thigh of a colt belonging to Mr. Millet of Rockhampton. This inflammation was followed by sores, which were dressed by the same persons who were employed ployed in milking. Soon after, all the cows and milkers in the dairy were affected with a disease, which appeared to be the genuine cow-pox.

In the second part of his Inquiry, p. 78, he alludes to that species of cow-pox which proceeds from simple inflammation; and, without pretending positively to decide, whether the matter thus produced in the cow possesses any specific property, like the former, he tells us, that his inquiries have not led him to adopt the supposition of its possessing such a specific property in any one instance.

In p. 90, he adduces some further reasons for the opinion he has adopted, that the cow-pox originates from the horse; an opinion which is general among the farmers in that part of the country. It is, indeed, the popular opinion, and particularly insisted upon by those who attend diseased cattle. He had frequently observed, that morbid matter, generated by the horse, casually communicated to the human subject, a disease so nearly resembling the cow-pox, that in many cases it was difficult to distinguish one from the other. He particularly confirms this idea by an extract of a letter from the Rev. Mr. Moore, of Chalford Hill, whose horse had the grease; which was soon followed by the cow-pox in the cow, and a similar disease in the servant.

In page 95, he observes, that from the similarity of local and constitutional symptoms, between the disease received from the cow and that received from the horse, the common people in his neighbourhood, through a strange perversion of terms, when infected by the horse, *frequently* call that disease the cow-pox. This shews, that it is a disorder of *frequent occurrence*; instead of being one that never occurs, as Dr. Kinglake supposes.

One instance which Dr. Jenner gives of this affection, was communicated to him by Mr. Fewster, of Thornbury, the celebrated inoculator for the small-pox, who is extremely well acquainted with the various appearances of the cow-pox, both in the brute animal and the human subject.

William Morris, of Almonsbury, applied to Mr. Fewster in April, 1798, informing him, that four days before he perceived a stiffness and swelling in both his hands; which were so painful, that it was with difficulty he continued his work. He had also been seized with a pain of the head, back, and limbs; and frequent chilly fits, succeeded by fever.

On

On examination, Mr. Fewster found him still affected with these symptoms, together with a great prostration of strength. His hands were chapped; and on the thumb of the right, there was a sore as large as a pea, and another on one of his fingers, which discharged an ichor. They were of a circular form; and he told Mr. Fewster, that at their first appearance, they resembled blisters occasioned by a burn. He complained of a very violent pain, extending up the arm to the axilla.

This disease was so similar to the cow-pox, that Mr. Fewster concluded it to have arisen from milking those animals; but the man assured him that he had not milked a cow for more than half a year; and that his master's cows had nothing the matter with them. Mr. Fewster then asked him, whether his master had any horse affected with the grease? to which he answered in the affirmative; adding, that he had constantly dressed him twice a day, for the last three weeks or more; and the smell of his hands was much like that of the horse's heels.

Three days afterwards, Mr. Fewster saw him again, and found him still complaining of pain, attended with the same febrile symptoms as before. The sores had now increased to the size of a seven-shilling piece; and there was one which Mr. Fewster had not observed before, on the forefinger of the left hand, as painful as those on the right. By proper applications they were healed in something more than a fortnight; but he lost all the nails of the thumb and fingers, which had been affected with this disease.

In the Medical Review for June, 1800, I published an account of Mr. Tanner, of Rockhampton, having successfully inoculated a cow with equine matter. Some doubt was entertained concerning the regularity of this experiment; but I am informed by the most respectable authority, that he has since again succeeded, in an experiment, concerning which there can be no doubt.

At the same time I published a case communicated to me by Dr. Marshall, previously to his going to the Mediterranean; which was as follows. Being consulted about the dairy-maid of a farmer in his neighbourhood, he perceived some pustules on the back of her hand. On inquiry he found, that the cow-pox was in the farm; and that the farmer's son, one morning, after dressing the heels of the horse, had milked the cow, because she was too unmanageable for the maid to milk her.

In the same paper I alluded to the evidence on this subject, lately received from Sir Christopher Pegge of Oxford, and Mr. Rankin of Eastbourne. The former was published in the Medical Review for November, the same year, in a letter to Dr. Jenner, and states, that he had derived his information from Mr. Lupton, a surgeon of Thame. Mr. Lupton's attention was first drawn to the subject in March 1800; when the son of Mr. Way, a farmer at Ichford, applied to him on account of a complaint in his hand, greatly resembling the cow-pox, both with regard to its local and constitutional symptoms. This complaint could not be accounted for from any other cause, than his washing the heels of a horse; as he had not milked a cow. Mr. Lupton was of opinion, that this disease was of the same nature with the cow-pox; and, after repeated inquiries, was convinced, that it is not the common grease in horses, which produces such effects in the cow, or the human subject.

On the 30th of the same month, Mr. Lupton had another patient, affected in the same manner, and from the same cause; Richard Hunt, a servant of Mr. Randolph, of Thame-Park Farm. His first symptoms were a stiffness and uneasiness of the arm, and a swelling of the axillary glands; succeeded by pustules on the hand, and a very painful one in the middle finger, of the blue appearance described by Dr. Jenner, as characteristic of the genuine disease.

These symptoms were attended with frequent rigors, heat, anxiety, giddiness, pain in the head and back, sickness and vomiting. He had a very bad night, and was rather delirious. The other arm also became stiff and painful. The next day red streaks appeared along the whole course of the lymphatics. On the 6th of April, the pustules on the hand had a dark-coloured depression in the centre, surrounded with an elevated margin of matter. As a proof that this man's complaint did not proceed from vaccine matter, he had not milked a cow for more than six months.

On the 9th of April, John Watson, another servant of Mr. Randolph, applied to Mr. Lupton, on account of a similar complaint. His particular employment was that of milking; but he had also assisted his fellow-servant in dressing the heels of the horse. The disorder shewed itself in the cows more than a week before it appeared on his hands; it is therefore doubtful whether he received the infection directly from the horse, or indirectly, from the cow.

cow. But, after the most minute investigation, Mr. Lupton is fully persuaded that it originated in the horse.

On the 18th of May, Sir C. Pegge was at Thame, and Mr. Lupton informed him, that Leonard Paling, a third servant of Mr. Randolph, was affected in the same manner as the two former. This man caught the disorder from the cows; never having assisted in dressing the horse.

Sir C. Pegge examined the case; and found the symptoms exactly correspond with those of the other persons, whose cases are already described. One of the axillary glands was still enlarged, and very tender; and his whole system had been much disordered, in consequence of the ulceration of his hand.

"Thus, says Sir Christopher Pegge, we have seen a complete series of facts, relative to the progress of this very important disease, as affecting the human frame; establishing its origin, in a disorder of the horse's heels, by farrier's termed a scratchy heel, and considered as widely different from common grease. The first servant was infected from the horse; the second conveyed the infection from the horse to the cows; and the third received it solely from the cows.

"From the last servant Mr. Lupton inoculated several children; some of whom I saw on the eighth day after inoculation, with the most decided appearance of the true cow-pox upon them. This appearance I could not mistake, after having witnessed so many instances of it at our friend's, Mr. Fermor, of Tusmore; whose benevolent and disinterested exertions have contributed so largely to the stock of facts, in support of a discovery, which, if the powerful argument of induction may be allowed, bids fair to be of the greatest benefit to mankind.

"One circumstance was remarkable in all the three persons above mentioned, namely, that they were affected with swellings in the axilla, and other symptoms denoting constitutional disease, before they had any ulceration or pain in their hands. Watson repeatedly assured me, that although both his hands were ulcerated, a swelling first appeared in the axilla of the left arm, followed by sickness, head-ach, &c. after which the ulceration commenced in the palm of the right hand, between the fore-finger and thumb."

Other avocations at present put it out of my power to add more on this subject; but it is my intention to resume it again. I cannot, however, omit this favourable opportunity of paying a sincere tribute of respect to the memory of that friend of humanity, of science, and of truth, Mr. Fermor, whose early and zealous exertions in vaccination I have elsewhere recorded. His general character is too well known, to require any encomium from my pen:

His saltem accumulem donis, et fungar inani

Munere. I am, &c.

15 *July*, 1806. JOHN RING.

